# Searching for target-specific and multi-targeting organics for Covid-19 in the Drugbank database with a double scoring approach

**DOI:** 10.1038/s41598-020-75762-7

**Published:** 2020-11-05

**Authors:** Natarajan Arul Murugan, Sanjiv Kumar, Jeyaraman Jeyakanthan, Vaibhav Srivastava

**Affiliations:** 1 Department of Theoretical Chemistry and Biology, School of Chemistry, Biotechnology and Health, KTH Royal Institute of Technology, 106 91 Stockholm, Sweden; 2 Division of Glycoscience, Department of Chemistry, School of Chemistry, Biotechnology and Health, KTH Royal Institute of Technology, Stockholm, Sweden; 3 Department of Bioinformatics, Alagappa University, Karaikudi, Tamilnadu India

**Keywords:** Medicinal chemistry, Theoretical chemistry, Drug discovery

## Abstract

The current outbreak of Covid-19 infection due to SARS-CoV-2, a virus from the coronavirus family, has become a major threat to human healthcare. The virus has already infected more than 44 M people and the number of deaths reported has reached more than 1.1 M which may be attributed to lack of medicine. The traditional drug discovery approach involves many years of rigorous research and development and demands for a huge investment which cannot be adopted for the ongoing pandemic infection. Rather we need a swift and cost-effective approach to inhibit and control the viral infection. With the help of computational screening approaches and by choosing appropriate chemical space, it is possible to identify lead drug-like compounds for Covid-19. In this study, we have used the Drugbank database to screen compounds against the most important viral targets namely 3C-like protease (3CLpro), papain-like protease (PLpro), RNA-dependent RNA polymerase (RdRp) and the spike (S) protein. These targets play a major role in the replication/transcription and host cell recognition, therefore, are vital for the viral reproduction and spread of infection. As the structure based computational screening approaches are more reliable, we used the crystal structures for 3C-like main protease and spike protein. For the remaining targets, we used the structures based on homology modeling. Further, we employed two scoring methods based on binding free energies implemented in AutoDock Vina and molecular mechanics—generalized Born surface area approach. Based on these results, we propose drug cocktails active against the three viral targets namely 3CLpro, PLpro and RdRp. Interestingly, one of the identified compounds in this study i.e. Baloxavir marboxil has been under clinical trial for the treatment of Covid-19 infection. In addition, we have identified a few compounds such as Phthalocyanine, Tadalafil, Lonafarnib, Nilotinib, Dihydroergotamine, R-428 which can bind to all three targets simultaneously and can serve as multi-targeting drugs. Our study also included calculation of binding energies for various compounds currently under drug trials. Among these compounds, it is found that Remdesivir binds to targets, 3CLpro and RdRp with high binding affinity. Moreover, Baricitinib and Umifenovir were found to have superior target-specific binding while Darunavir is found to be a potential multi-targeting drug. As far as we know this is the first study where the compounds from the Drugbank database are screened against four vital targets of SARS-CoV-2 and illustrates that the computational screening using a double scoring approach can yield potential drug-like compounds against Covid-19 infection.

## Introduction

The viral infection affects all forms of life including birds, mammals and humans causing severe effects on the healthcare system and economy. Most of the viral infections are contagious and spread through air, water, food or exchange of body fluids. The viral infection can lead to outbreak, or can become epidemic or pandemic based on the spreading mechanism and strength of virus-host cell interaction. There are many factors that dictate the severity of a viral infection such as intrinsic pathogenicity, mortality rate, basic reproduction number^1^. It appears like the ongoing pandemic due to SARS-CoV-2 is the one of the most adverse infections observed during recent decades. Even though the mortality rate appears to be quite lower for this compared to previous outbreaks due to SARS and MERS viruses, the severity, disruption to the healthcare and damage to the economy have become very high due to its more aggressive human-to-human spread^2^. After its first report in December, 2019 within 6 months time, it has spread to almost all countries, infecting more than 44 M people. Moreover, it has been lethal to more than 1.1 M people. It is a challenging time for all researchers in medicine and pharmacology to develop a vaccine, small molecular drug or an epitope to circumvent the current case of outbreak^3^. There exist standard protocols to rationally develop such medicine from scratch for any such infections starting from gene mining which involves targets discovery and identification of lead drug-like compounds from structure based design. Another approach is through the high throughput experimental screening^4^ of compounds from chemical space which is rather shooting for something in the darkness. Our aim here is to present a rational approach involving computational screening for identification of lead drug-like compounds for Covid-19 associated coronavirus^5^.

The genomics data encode the biomolecular machineries necessary for the life processes of any organism including pathogens and can be used to obtain information regarding potential targets relevant for therapy or diagnostics^6^. Therefore, in order to design drugs against viral pathogens, we need to start with the data mining of viral genome. More than 10,000 genomics data are already deposited for SARS-CoV-2 in GISAID, an open source online platform (https://www.gisaid.org)^7^. It is a wealth of information which can be used to find the routes followed by the virus to spread the infection^8^. The SARS-CoV-2 genome is made of less than 30000 nucleotides and contains genes for 29 different proteins^9^. The ORF1ab alone encodes as many as 16 non-structural proteins^10^. Some of the key proteins encoded by this gene are PLpro (NSP2), 3CLpro (NSP5), RdRp (NSP12), and helicase (NSP13) which play a vital role in the replication and transcription^11,12^. The ORF2-10 encodes various structural proteins such as membrane protein (M), envelope protein (E), spike protein (S), nucleocapsid protein (N) and other auxiliary proteins^12,13^. The M, E and S make the viral coat while the RNA gene is packaged within the N protein^11,12^. Further, the spike protein is involved in the host cell recognition and in particular binds to Angiotensin-converting enzyme 2 (ACE2) mammalian receptor^14^. Based on their involvement in different biological processes, many SARS-CoV-2 proteins (e.g. spike protein, PLpro, 3CLpro, RdRp, and helicase etc) can be considered as potential targets for therapy. More specifically, for therapeutic purpose, it is essential to target enzymes involved in viral replication as well as transcription. In addition, we need to target those catalytic sites involved in the key enzymatic reaction. In SARS-CoV-2, the main role of 3CLpro and PLpro proteases is to cleave the polyprotein into smaller functional units to facilitate replication/transcription process and thus are potential targets for the therapeutics^15^.

Designing and screening of small molecules or peptides targeting these vital proteins can help in developing therapeutics against the infection. High-throughput experimental screening on viral particles or specific target could be performed to test the activity of such compounds, however, this is expensive and time taking. In addition, extreme care needs to be taken as it may require handling of live and potentially pathogenic viral strains. In this study, we used an alternative approach by computational screening of compounds from the Drugbank database^16^ against the selected viral drug targets using a molecular docking approach^17,18^. The Drugbank database is a chemical space of compounds approved by FDA and molecules under drug trials (investigational and experimental). Since these compounds are already under clinical trials or in the market, the synthesizability^19^ is not a problem which is often a major problem when the compounds are designed using *de novo* design^20^ or those designed from fragments based drug discovery approaches^21^. Further since many of the compounds are approved, if they are found to be active against viral targets during the computational screening they can be straight-away repurposed for treating Covid-19^22^. Since these “approved” compounds are already verified for safety, the time associated with the clinical trials can be significantly reduced. The structure based screening of compounds requires 3 dimensional structure for the viral proteins. As of now, the structures for 3CLpro, spike glycoprotein, ADP ribose phosphatase, RNA binding domain of nucleocapsid phosphoprotein, Endoribonuclease have been reported in the protein databank (PDB)^23^. The spike protein structure is based on cryogenic electron microscopy and has been reported in two different conformations namely when it is bound to ACE-2 mammalian receptor and in free state^24,25^. In case of 3CLpro, the crystal structures are reported for both apo form and for the co-crystallized form with certain inhibitors^26^. For computational screening, we have used the crystal structures for the two targets 3CLpro and spike protein. For the remaining two targets PLpro and RdRp, we have carried out homology modeling using template structures from SARS-CoV-1 which was reported to have a highly homologous genome as SARS-CoV-2. The models were developed using SWISS-MODEL, a web server for doing homology modeling^27^.

For each of the selected target protein, we identified top five compounds from a list of “approved” drugs and another top five from the subset of “investigational” drugs. Further the stability and binding affinity of these screened compounds have been validated using molecular dynamics and molecular mechanics-generalized Born surface area (MM-GBSA) approach^28^. Even though the molecular docking approaches as implemented in Autodock, AutoDock Vina and Glide are quite fast, they were shown to fail in ranking complexes in many occasions^29^. Among many force-field based scoring functions, the MM-GBSA based ranking of protein-ligand complexes has been reported to be reliable often^29–31^. This has been the reason for choosing a double-scoring approach based on AutoDock Vina and MM-GBSA based binding free energies in this study. In addition to identifying the lead drug-like molecules for various targets, the study proposes the drug molecules which can target multiple targets simultaneously^32^. The study also involves the computational validation of many drug compounds currently considered for drug trials^33^ in various countries by estimating binding free energies using MM-GBSA approach. The trial compounds included in this study are Remdesivir, Chloroquine, Lopinavir, Oseltamivir, Ritonavir, Favipiravir, Baricitinib, Darunavir and Umifenovir^34–36^. We also would like to recall that many researchers have screened compounds from different chemical-spaces such as natural products database^37^, FDA-approved compounds^38^, phytochemicals^39–41^, marine natural products^42^ ChEMBL^43–45^ and pubchem^44^ databases against different viral targets by using different scoring functions^46^. The main difference of the current study is that we have considered four different viral targets and so we are able to propose drug cocktails effective for Covid-19 along with suggestions of drugs which have the potential as multi-targeting drugs. Further we have also discussed on the computational scoring of trial compounds in a way to argue whether we need to validate the compounds before considering for drug trials by employing cost-effective screening approaches.

## Results and discussion

In this study, we have identified several compounds from the Drugbank that are predicted to bind to individual target proteins with high affinity (Tables 1, 2 and 3). In addition, we also identified compounds that potentially interact with two or more viral proteins (Table 4). In the context of SARS-CoV-2 viral therapeutics^32,47^, binding of a drug molecule to a single or multiple targets can be of significance depending upon the different stages of the viral infection. For instance, during the host cell recognition only the spike protein of SARS-CoV-2 plays a key role and can be targeted. However, once the infection occurs, other proteins associated with transcription and replication processes are expressed. In this case, it is advantageous to use a drug which targets multiple proteins or cocktail of drugs with each drug having significant binding affinity towards a specific target. Due to the complexity involved in the development of infection, it is desirable to target multiple targets with many low affinity ligands instead of targeting a single target with high affinity ligand^47^. This also has an advantage that even when a specific target mutates rapidly, the other targets can be inhibited by the drug cocktails which eventually makes the treatment effective. Such combination of drugs subscription is already in practice for viral infections^48^. In case of HIV treatment, a combination of drugs belonging to categories such as nucleoside reverse transcriptase inhibitor, non-nucleoside reverse transcriptase inhibitor, protease inhibitor and integrase inhibitor has been successfully tested^49^. For example, a FDA-approved drug Combivir is a mixture of AZT and 3TC and targets enzymes which appear in the early and later stage of HIV replication^48^.

**Table 1:** The compounds selected from the Drugbank database based on their binding affinity for the four vital targets in Covid-19.

3CLpro	PLpro	RdRp	Spike
**Approved**			
Olaparib ($-$ 9.2)	Tadalafil ($-$ 9.2)	Lumacaftor ($-$ 9.9)	Dexamethasone metasulfobenzoate ($-$ 10.4)
Baloxavir marboxil ($-$ 8.9)	Metocurine($-$ 9.0)	Ergotamine ($-$ 9.4)	Nilotinib ($-$ 9.9)
Entrectinib, (− 8.7)	Lorlatinib ($-$ 9.0)	Natamycin($-$ 9.4)	Sonidegib ($-$ 9.8)
Dexamethasone metasulfobenzoate ($-$ 8.7)	Lumacaftor ($-$ 8.9)	Dihydroergotamine ($-$ 9.3)	Enasidenib ($-$ 9.8)
Tadalafil ($-$ 8.7)	Natamycin ($-$ 8.8)	Imatinib ($-$ 9.3)	Regorafenib, Lifitegrast, Capmatinib ($-$ 9.7)
**Investigational**			
LY-2090314 ($-$ 10.3)	Zoliflodacin ($-$ 9.8)	Phthalocyanine ($-$ 10.6)	Lifirafenib ($-$ 10.7)
10-hydroxy camptothecin ($-$ 9.3)	JE-2147 ($-$ 9.7)	RU85053 ($-$ 9.9)	Resiniferatoxin ($-$ 10.6)
Tivantinib ($-$ 9.0)	Phthalocyanine ($-$ 9.6)	Laniquidar ($-$ 9.9)	JTK-853 ($-$ 10.6)
Lurtotecan ($-$ 9.0)	Quarfloxin ($-$ 9.5)	CD564 ($-$ 9.8)	Tegobuvir ($-$ 10.5)
Zk-806450 ($-$ 8.9)	CP-609754 ($-$ 9.5)	Golvatinib ($-$ 9.8)	PCO-371 ($-$ 10.5)

The binding affinities are given in kcal/mol.

**Table 2:** The compounds from the Drugbank database and the corresponding binding free energies towards the four vital targets in SARS-CoV-2.

3CLpro	PLpro	RdRp	Spike
$-$ 43.6 (Baloxavir marboxil)	$-$ 35.9 (Natamycin)	$-$ 43.1 (RU85053)	$-$ 46.4 (Sonidegib)
$-$ 43.2 (LY$-$ 2090314)	$-$ 35.0 (Lumacaftor)	$-$ 36.1 (Golvatinib)	$-$ 44.5 (Regorafenib)
$-$ 31.6 (Entrectinib)	$-$ 27.5 (CP-609754)	$-$ 29.5 (Natamycin)	$-$ 42.4 (Lifitegrast)
$-$ 30.2 (Tadalafil)	$-$ 23.7 (Zoliflodacin)	$-$ 27.2 (Lumacaftor)	$-$ 40.4 (PCO$-$ 371)
$-$ 27.6 (Tivantinib)	$-$ 22.6 (Quarfloxin)	$-$ 18.6 (Dihydroergotamine)	$-$ 36.2 (Resiniferatoxin)

The binding free energies are given in kcal/mol. The free energies are computed using MM-GBSA approach as an average over 500 configurations extracted from molecular dynamics trajectories.

**Table 3:** Various contributions to the binding free energies of selected high affinity compounds for various viral targets.

Site	$\Delta E_{vdw}$	$\Delta E_{elec}$	$\Delta G_{GB}$	$\Delta G_{SA}$	$\Delta G_{binding}$
**3CLpro**					
Baloxavir marboxil	$-$ 53.8	$-$ 7.0	23.3	$-$ 6.0	$-$ 43.6
LY$-$ 2090314	$-$ 49.5	$-$ 19.0	30.7	$-$ 5.4	$-$ 43.3
**PLpro**					
Natamycin	$-$ 43.2	$-$ 40.9	53.6	$-$ 5.4	$-$ 35.9
Lumacaftor	$-$ 44.2	$-$ 9.2	24.0	$-$ 5.5	$-$ 35.0
**RdRp**					
RU85053	$-$ 66.0	$-$ 38.2	70.0	$-$ 8.7	$-$ 43.1
Golvatinib	$-$ 49.6	$-$ 33.4	53.3	$-$ 6.5	$-$ 36.1
**Spike protein**					
Sonidegib	$-$ 61.5	$-$ 26.5	49.2	$-$ 7.6	$-$ 46.4
Regorafenib	$-$ 54.9	$-\,$31.6	49.1	$-$ 7.1	$-$ 44.5

The free energies were computed using MM-GBSA approach.

**Table 4:** Multi-targeting drugs for SARS-CoV-2. The compounds are identified based on the binding free energies computed using AutoDock Vina for the three viral targets namely 3CLpro, PLpro and RdRp. The binding energies are in kcal/mol.

Drug	3CLpro	PLpro	RdRp
DB04016	$-$ 8.8	$-$ 9.4	$-$ 9.5
Phthalocyanine	$-$ 8.8	$-$9.6	$-$ 10.6
DB08386	$-$ 8.7	$-$ 8.8	$-$ 9.1
Tadalafil	$-$ 8.6	$-$ 9.2	$-$ 9.1
Lonafarnib	$-$ 8.5	$-$ 8.5	$-$ 9.1
Nilotinib	$-$ 8.4	$-$ 8.7	$-$ 9.1
Dihydroergotamine	$-$ 8.3	$-$ 8.5	$-$ 9.3
R-428	$-$ 8.3	$-$ 8.7	$-$ 9.4

### Potential compounds from the Drugbank database against the four key viral target proteins

Based on the larger binding affinity towards each of the selected Covid-19 targets i.e. 3CLpro, PLpro, RdRp, spike-protein, top five FDA-approved compounds were selected from the Drugbank database (Table 1). In addition, we also selected another top five compounds showing higher binding affinities which are in the investigational/experimental stage at the moment (Table 1). In general, it was observed that the compounds belonging to the latter category have higher binding affinity as compared to the FDA-approved compounds (Table 1). This may be due to significantly higher number of compounds in the investigational category as compared to the subset of FDA-approved compounds in the Drugbank database. It is also supported by the fact that the use of a larger chemical space, will result in identifying compounds with better binding affinity and specificity towards specific targets.

#### 3CLpro inhibitors

The selected top five FDA-approved compounds showing high affinity for 3CLpro protein are Olaparib, Baloxavir marboxil, Entrectinib, Dexamethasone metasulfobenzoate and Tadalafil (Table 1). The binding affinities are in the range of $-$ 8.7 to $-$ 9.2 kcal/mol (Table 1). In this list, Olaparib and Entrectinib are anticancer compounds while Baloxavir marboxil is an antiviral compound shown to be active against influenza A and B viruses. Even though the Drugbank database contains many antiviral compounds, only Baloxavir marboxyl (BM) showed high score against 3CLpro target of SARS-CoV-2. It is worth mentioning that this compound is being considered for drug trials along with other antiviral compounds such as Oseltamivir and Umifenovir by the company Shionogi, Toyama Chemical^33^. Drug repurposing is considered as an expeditious approach to find potentially active compounds against Covid-19 associated viral targets and the advantage is that these compounds have favourable ADME/T properties. We also selected other five compounds showing high binding affinity towards 3CLpro and are listed under the investigational category in the Drugbank database (Table 1). The compounds are LY-2090314, 10-hydroxycamptothecin, Tivantinib, Lurtotecan and Zk-806450. These compounds have binding free energies in the range of $-$ 8.9 to $-$ 10.3 kcal/mol with LY-2090314 with the best binding affinity. Some of these compounds are reported to have anticancer (LY-2090314, Tivantinib), and antineoplastic (10-hydroxycamptothecin, Lurtotecan) properties^16^.

**Figure 1:**
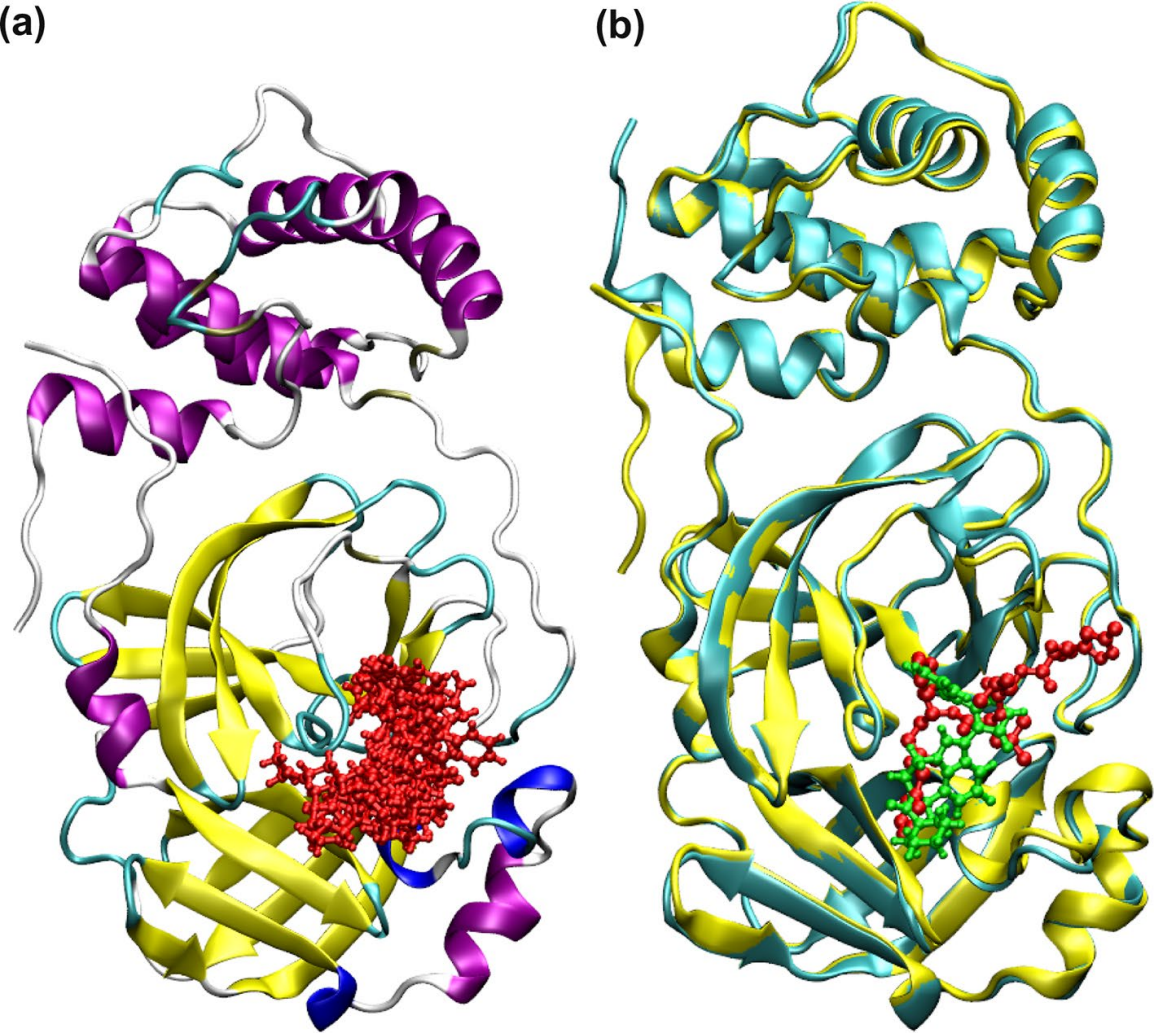
(**a**) The spatial overlap of binding modes for various high affinity compounds for 3CLpro. The ligands having binding free energies less than $-$ 9.0 kcal/mol were chosen. (**b**) Comparative binding mode of the best binder with that of N3 inhibitor (shown in red color).

Since the crystal structure of 3CLpro in complex with an irreversible inhibitor N3 is already available, information about the binding site responsible for carrying out the enzymatic reaction is known. In the Fig. 1a, the binding modes of the top 10 compounds listed in Table 1 and discussed above are shown. Different secondary structures such as helix (black), 3–10 helix (magenta), beta-sheet (yellow), coil-like (white) structures in the target are depicted using the default VMD^50^ color code specification. Comparative binding mode of LY-2090314 with N3 inhibitor is shown in the Fig. 1b. It is clear that all compounds bind to the substrate binding site which indicates that they can exert therapeutic activity by inhibiting the replication role of this enzyme. It is worth mentioning that the N3 inhibitor is irreversible as it forms a covalent bond with CYS145 residue of the target protein^51^. In contrary, all of the compounds studied here are reversible inhibitors.

#### Papain-like protease inhibitors

The top five FDA-approved compounds showing high affinity towards the protein PLpro, are Tadalafil, Metocurine, Lorlatinib, Lumacaftor, Natamycin while the compounds identified from the “investigational” category are Zoliflodacin, JE-2147, Phthalocyanine, Quarfloxin and CP-609754. The binding free energy range for the former set of compounds is between $-$ 8.8 and $-$ 9.2 kcal/mol (Table 1) whereas for the latter between $-$ 9.5 and $-$ 9.8 kcal/mol (Table 1). The Zoliflodacin, a spiropyrimidinetrione type antibiotic compound is in the phase-II clinical trials for the treatment of *Neisseria gonorrhoeae* infection. It acts on the type II topoisomerases and inhibits DNA biosynthesis in bacteria^52^. Interestingly, JE-2147 is a dipeptide based antiviral compound which acts on Gag-Pol polyprotein of HIV and is showing promising potency against multi-PI resistant HIV^53^. The compound phthalocyanine is an investigational drug used for photodynamic therapy of actinic keratosis, Bowen’s disease, skin cancer, or stage I or stage II Mycosis Fungoides^16^. The compound quarfloxin is also an anticancer compound (leukemia). The compound CP-609754 is under phase-I trial for treating advanced malignant tumours and exhibits its activity as farnesyltransferase inhibitor^54^. Among approved compounds, Tadalafil, Metocurine, Lorlatinib and Lumacaftor are respectively used for treating erectile dysfunction, muscular relaxant, ALK-positive metastatic non-small cell lung cancer and cystic fibrosis. Only Natamycin is an antibiotic and inhibits fungal growth by binding to sterols (ergosterol in particular)^55^.

**Figure 2:**
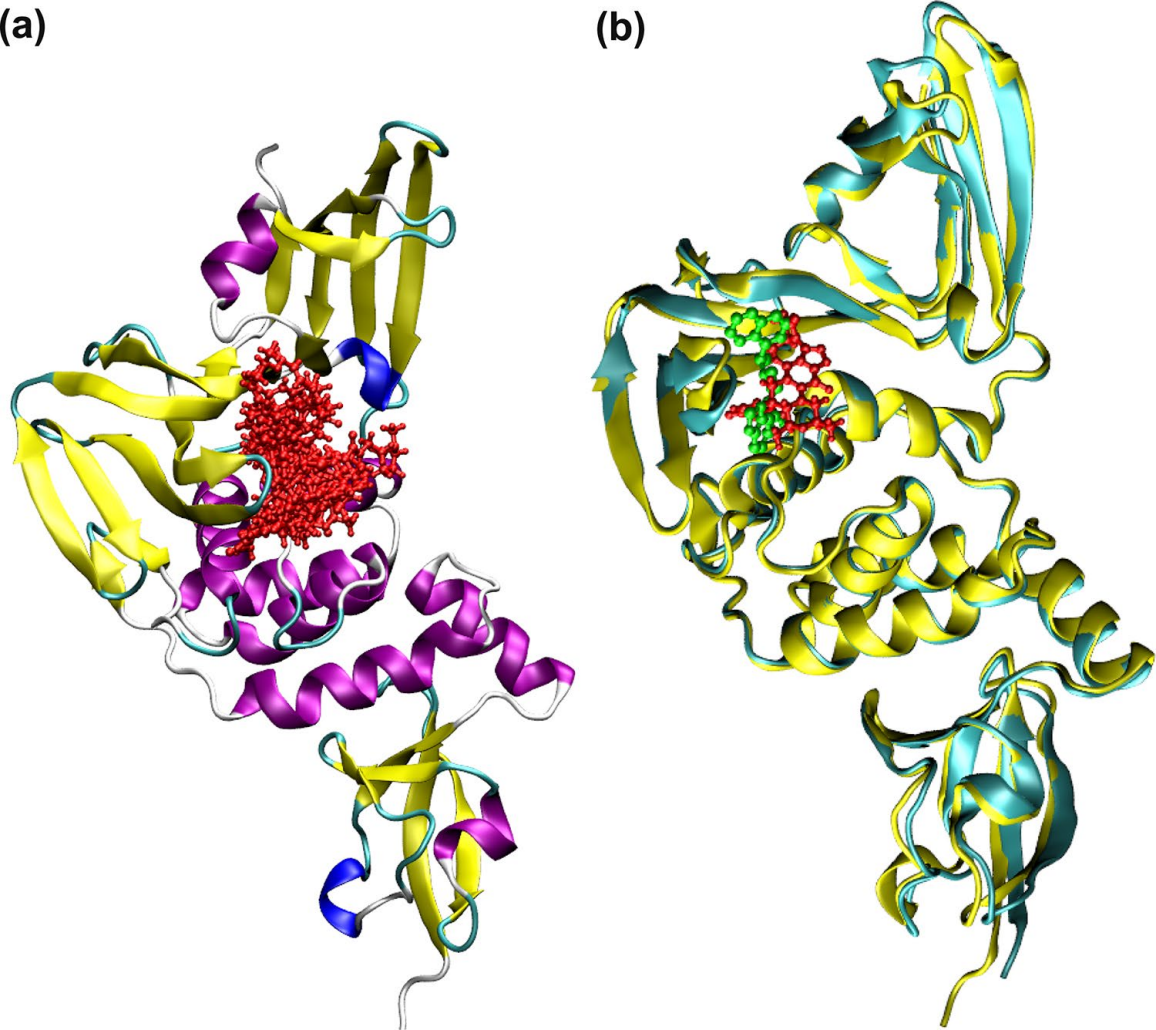
(**a**) The spatial overlap of binding modes for various high affinity compounds for PLpro. The ligands having binding free energies less than $-$ 9.0 kcal/mol were chosen. (**b**) Comparative binding mode of the best binder with that of inhibitor N-(1,3-benzodioxol-5-ylmethyl)-1-[(1R)-1-naphthalen-1-ylethyl]piperidine-4-carboxamide (GRM) of PLpro enzyme of SARS-CoV-1 based on 3MJ5 crystal structure. GRM is shown in green color.

The PLpro also has well defined binding site as shown in the Fig.  2a. All the 10 compounds listed in Table 1 bind to this catalytic site and thus can be potentially used as drug molecules against Covid-19 infection. In the Fig. 2b, we show that the compound Zoliflodacin binds at the same substrate binding site as the inhibitor GRM of PLpro in SARS-CoV-1.

#### RNA dependent RNA polymerase inhibitors

**Figure 3:**
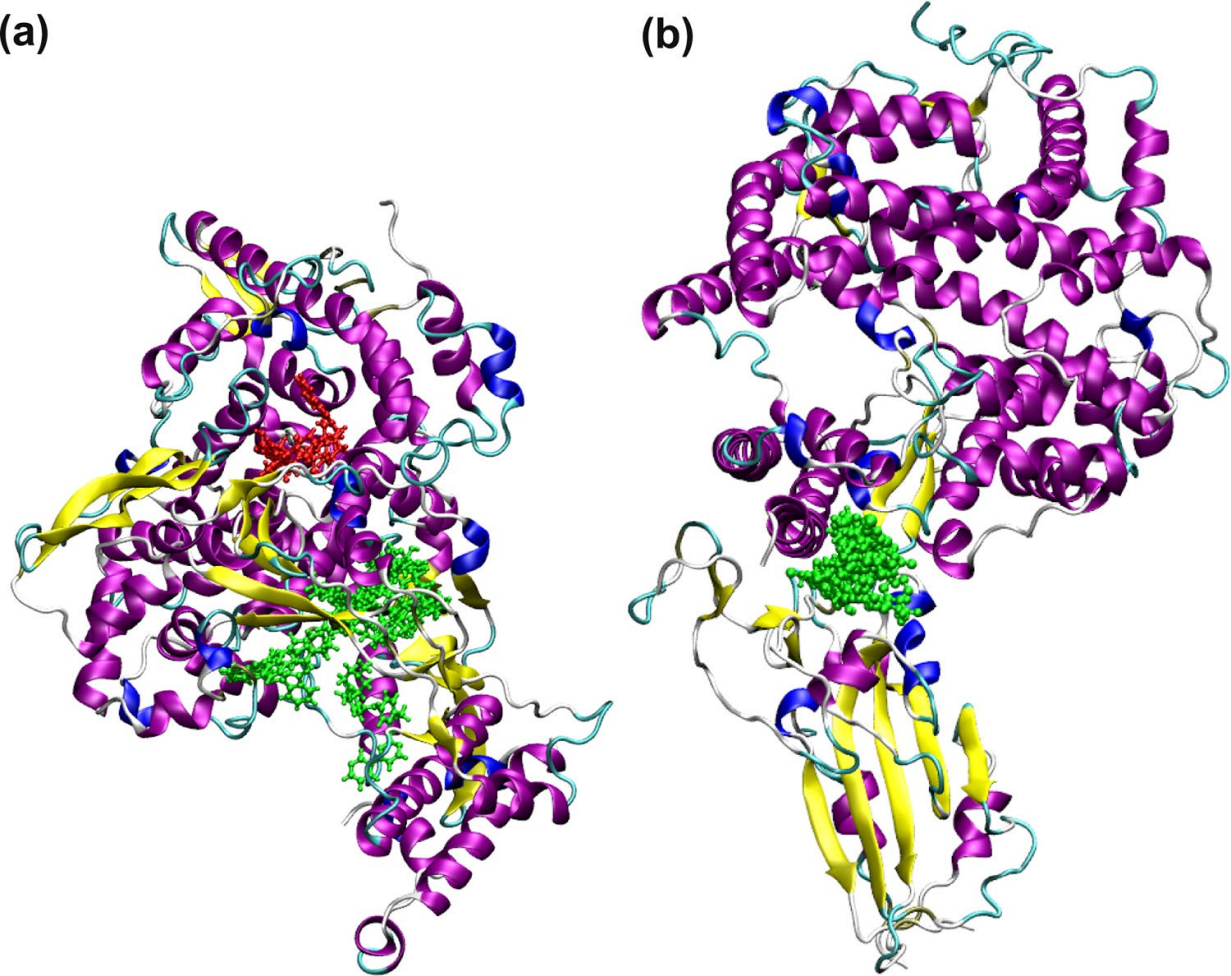
(**a**) The spatial overlap of binding modes for various high affinity binders for RdRp target. The ligands having binding free energies less than $-$ 9.0 kcal/mol were chosen. As can be seen except the RU85053 and CD564 (shown in red color), the rest of the drugs (shown in green color) target nucleotide binding domain. (**b**) The spatial overlap of binding modes for various high affinity binders in the interfacial region of spike protein (receptor binding domain) and ACE-2 mammalian receptor. The ligands having binding free energies less than $-$ 9.7 kcal/mol were chosen.

In the case of RdRp binders belonging to the category of investigational drugs, the phthalocyanine is a photodynamic drug against certain types of cancer, while Laniquidar and golvatinib are active against breast cancer and carcinoma^16^. The pharmacological action of the other two selected compounds RU85053 and Golvatinib is not documented elaborately in the literature. The approved list of high affinity compounds for RdRp protein includes Lumacaftor, Ergotamine, Natamycin, Dihydroergotamine and Imatinib. We have already discussed the pharmacological properties of the compounds Lumacafor and Natamycin in the previous section as they were also predicted to interact with PLpro (Table 1). The compound, Imatinib is an anticancer drug and is used for the treatment of chronic myelogenous leukemia (CML), gastrointestinal stromal tumors and acts by inhibiting tyrosine kinase enzyme^16^. Both Ergotamine and Dihydroergotamine are used for treating the migraine disorders. Figure 3a shows the binding modes for all 10 compounds related to RdRp protein (Table 1). Except for the two compounds i.e. RU85053 and CD564 all other compounds bind to the nucleotide binding site. The therapeutic role of these compounds is associated with their interference with the nucleotide binding to this target.

#### Compounds binding to spike protein

The subset of approved compounds which bind to interfacial region located between the viral spike protein and ACE-2 mammalian receptor with high binding affinity are Dexamethasone metasulfobenzoate (DMS), Nilotinib, Sonidegib, Enasidenib, Regorafenib, Lifitegrast and Capmatinib. The binding affinities for this set of compounds are in the range $-$ 9.7 to $-$ 10.4 kcal/mol. In particular, last three compounds have the same binding affinity of $-$ 9.7 kcal/mol. Among these compounds, the pharmacological property of DMS is not documented well in the literature and it appears to be used in the treatment of anaesthetic complication, nausea and vomiting^16^. The compound Nilotinib is in practice to treat chronic myelogenous leukemia that is resistant to Imatinib (which we reported as RdRp inhibitor in the above subsection). The compounds, Sonidegib and Enasidenib are again anticancer compounds used in the treatment of basal cell carcinoma and acute myeloid leukemia respectively while Regorafenib is active against metastatic colorectal cancer and advanced gastrointestinal stromal tumours. The compound, Capmatinib is used in the treatment of melanoma, gliosarcoma, solid tumors, colorectal cancer. The remaining compound in the subset, Lifitegrast is active against dry eye syndrome. The top most high affinity compounds in the subset of experimental drugs include Lifirafenib, Resiniferatoxin, JTK-853, Tegobuvir and PCO-371 and the binding affinities are in the range $-$ 10.5 to $-$ 10.7 kcal/mol. The top compound, Lifirafenib is also an anticancer agent against advanced or metastatic malignant solid tumors. The compound Resiniferatoxin is a pain reliever while JTK-853 is potent against Hepatitis C virus infection. Interestingly, Tegobuvir is a non-nucleoside inhibitor against HCV RNA replication and potent investigational compound for the treatment of chronic HCV infection^56^. The compound PCO-371 was an investigational compound for treating hypoparathyroidism and works as a parathyroid 1 receptor agonist.

**Figure 4:**
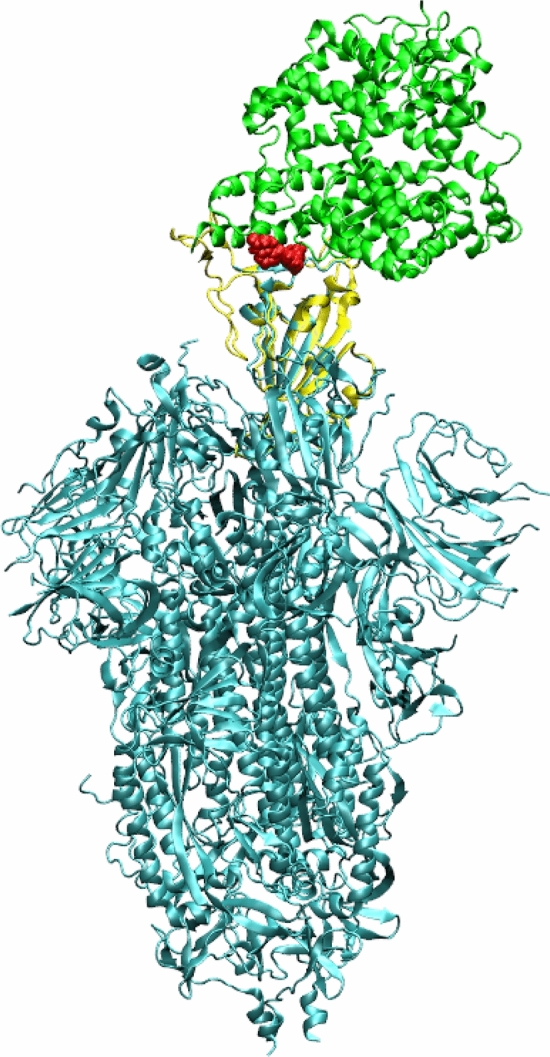
The structure of full spike protein (shown in cyan color) and ACE-2 (shown in green color) complex with a ligand (shown in red color) bound to the interfacial region of these two proteins. The receptor binding domain of spike protein is shown in yellow color.

As mentioned earlier, the viral spike protein recognizes the mammalian ACE-2 receptor and mediates the binding to the host cell membrane. The drug-like compounds should weaken the protein–protein interaction through binding to interfacial region between the spike protein and ACE-2. Indeed, the compounds studied here bind to a site which is located in the interfacial region between the two biomolecules (refer to Fig. 3b). However, it is difficult to say whether the binding of these compounds will increase or decrease the interaction between the two biomolecules i.e. spike protein and ACE-2 receptor. If the ligands bind to both biomolecules with stronger binding affinity then the protein–protein interaction will be increased. However, if it binds to any one of the biomolecules with higher binding affinity, then the protein–protein interaction can be weakened and only this situation will have expected therapeutic effect. Figure 4 shows the full spike protein and ACE-2 complex with a ligand bound to interfacial region between these two proteins.

### Binding affinities of the compounds from the Drugbank database

**Figure 5:**
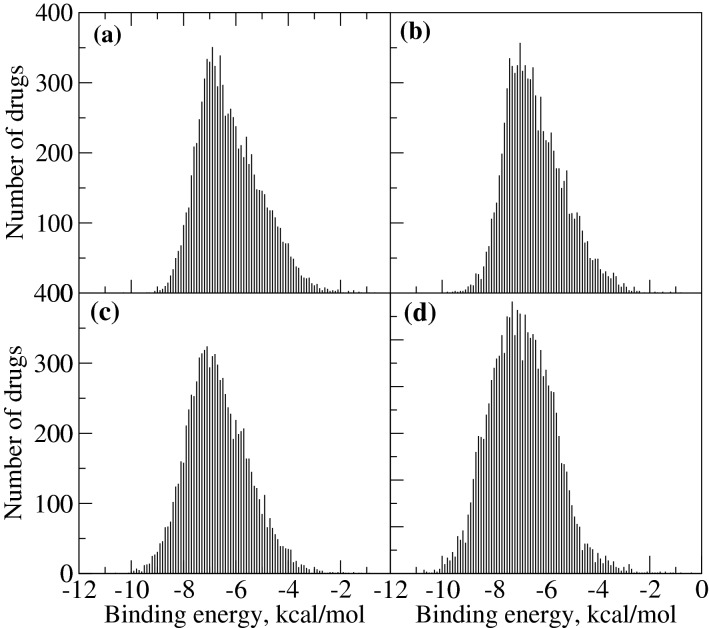
Distribution of binding energies of compounds in the Drugbank database towards the four viral targets, (**a**) 3CLpro, (**b**) PLpro, (**c**) RdRp (**d**) spike protein-ACE-2 complex.

In order to check the total number of lead compounds which can be identified with the use of AutoDock Vina from the Drugbank database, we calculated the binding energy distributions for all molecules in this database (8773 compounds with 3D structure) in four different viral targets (Fig. 5). Most of the compounds are reported to have a significant to considerable binding affinity in the range of $-$ 4 to $-$ 9 kcal/mol. A careful analysis of the plot shows that the number of compounds having binding affinity < $-$ 9 kcal/mol vary depending upon the target. The spike protein was found with the most number of compounds followed by the RdRp. In addition, both proteases have comparable numbers of compounds with the binding affinity lower than this value. This can be directly related to the binding site volume and molecular volume of lead-compounds. Since the spike protein and RdRp have larger binding sites, most of the drug molecules can bind to these targets without any restriction on their size. If we are looking for compounds having the binding affinity in the subnanomolar range (which corresponds to binding free energies < $-$ 12.35 kcal/mol), the Drugbank database does not have any compounds to offer and other chemical spaces such as ZINC^57^ or GDB13^58^ can be exploited to look for such compounds. Another option is to modify the chemical structure of the top compounds that were obtained from our screening in a way to maximize the interaction with neighboring residues in the binding site. We have analysed the compounds having binding affinity in the nanomolar range (i.e. 1–100 nM which corresponds to binding free energies < $-$ 9.61 kcal/mol). In particular, for 3CLpro, there is just one compound (LY-2090314 ) and for PLpro, there are two compounds (Zoliflodacin and JE-2147) with binding affinity in this range. In the case of RdRp, there are 16 such compounds (refer to Table 1 for top 5 of them) and for spike protein 51 such compounds (refer to Table 1 for the top 5 of them). Altogether, based on this observation, we can conclude that the Drugbank database does not have many compounds with superior binding affinity for both proteases (3CLpro and PLpro) however, for the other two targets (RdRp and S protein) reasonable number of such compounds are present.

The AutoDock Vina based scoring uses a single rigid protein conformation for ranking the compounds. However, inclusion of the conformational flexibility of protein might help to remove strains for certain ligands in the binding site and so a rescoring with the inclusion of protein-ligand dynamics will result in an improved ranking of the ligands. Therefore, we have further ranked the 10 compounds (which include both approved and investigational compounds) in Table 1 using MM-GBSA based binding free energies. In this case, the binding free energy calculations were carried out for the protein-ligand configurations (500 in total) picked up from the molecular dynamics simulations and allowed full conformational flexibility for both proteins and ligands. The binding free energies were computed as the sum of van der Waals, electrostatic, polar and non-polar solvation energies where the latter two terms are computed using an implicit solvent model.

### Rescoring the compounds using MM-GBSA based binding free energy calculations

The top 5 compounds for each target obtained after rescoring all 10 compounds (Table 1) using the MM-GBSA based calculations are shown in Table 2. We found that the ranking for the compounds is different when compared to the AutoDock Vina based scoring which has to be attributed to lack of protein conformational sampling in the latter case. For the 3CLpro target, the latter scoring approach produced LY-2090314 and 10-hydroxycamptothecin as the top high affinity compounds. However, the MM-GBSA based scoring yielded Baloxavir marboxil and LY-2090314 as the top high affinity compounds. Similarly, for PLpro, AutoDock Vina predicted Zoliflodacin and JE-2147 as the top 2 inhibitors while Natamycin and Lumacaftor were the top compounds predicted with MM-GBSA based scoring. In the case of RdRp, RU85053 and Niltinib are the two top performing compounds and Sonidegib and Regorafenib are the compounds identified for the spike protein. Another striking observation was regarding the larger variation in binding free energies computed from the MM-GBSA approach. Binding free energies vary over a range of $-$ 27.6 to $-$ 43.6 kcal/mol in the case 3CLpro associated inhibitors. The underestimation of binding free energies by MM-GBSA approach has also been shown in the literature^59^. Nevertheless, it is considered to be a better approach in ranking complexes when compared to AutoDock or AutoDock Vina^29,59^. For ranking of complexes, relative difference in free energies is more significant compared to the absolute binding free energies and this makes the MM-GBSA based approach promising. Furthermore, we also checked various contributions to the total binding free energies obtained using MM-GBSA approach for the top 2 compounds in each target (refer to Table 3). The following observations were made: (i) major contributions to total binding free energies are due to $\Delta E_{vdw}$ term which suggests that the complexation is driven mostly by the van der Waals type interactions. (ii) the total electrostatic contributions which is a sum of electrostatic interaction between the target-ligand ($\Delta E_{elec}$) and polar solvation energies ($\Delta E_{GB}$) are acting against the complexation (Table 3). We learn that the compounds which are engaged in larger van der Waals interactions with targets are the best inhibitors and so the compounds with more number of hydrophobic groups are preferred. Since the electrostatic interactions between the polar functional groups and charged residues in protein are nullified by polar solvation energies, the compounds with hydrophilic groups are less favoured as inhibitors.

**Figure 6:**
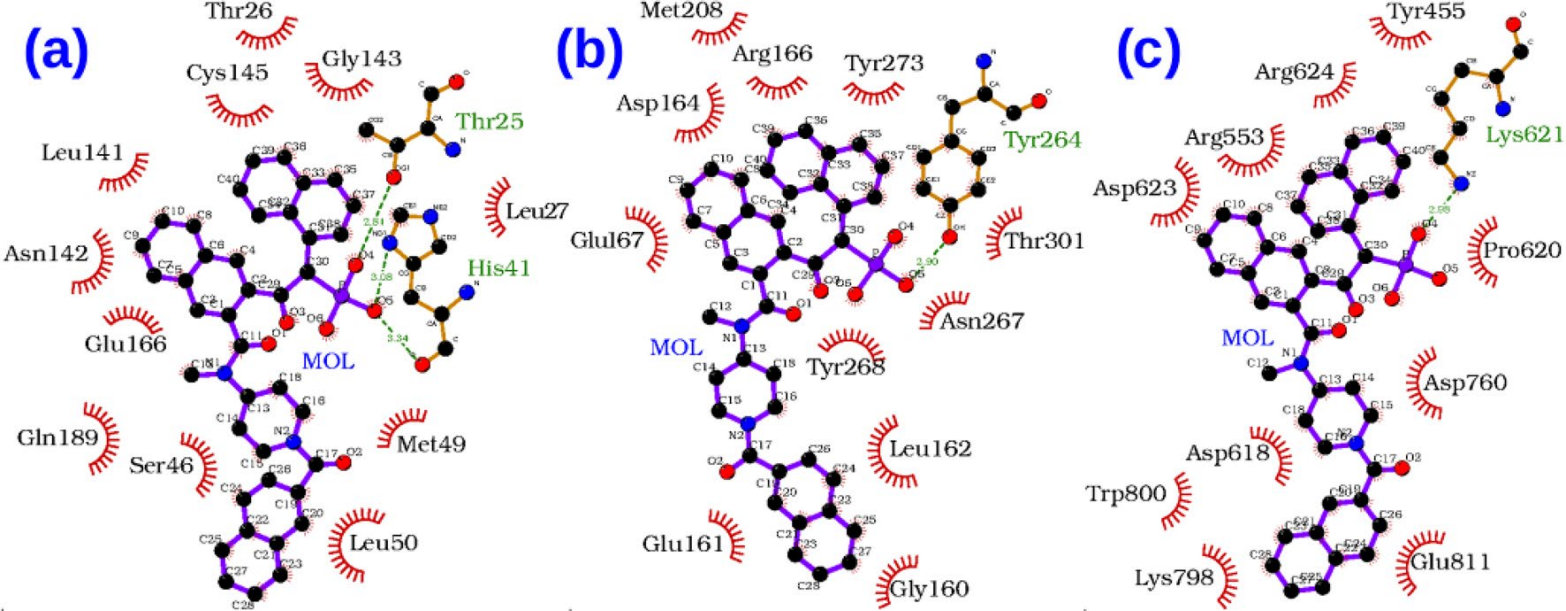
Protein-ligand interaction diagrams for the compound, DB04016 with three targets (**a**) 3CLpro, (**b**) PLpro, (**c**) RdRp respectively.

### Multi-targeting drugs from the Drugbank database for Covid-19

**Figure 7:**
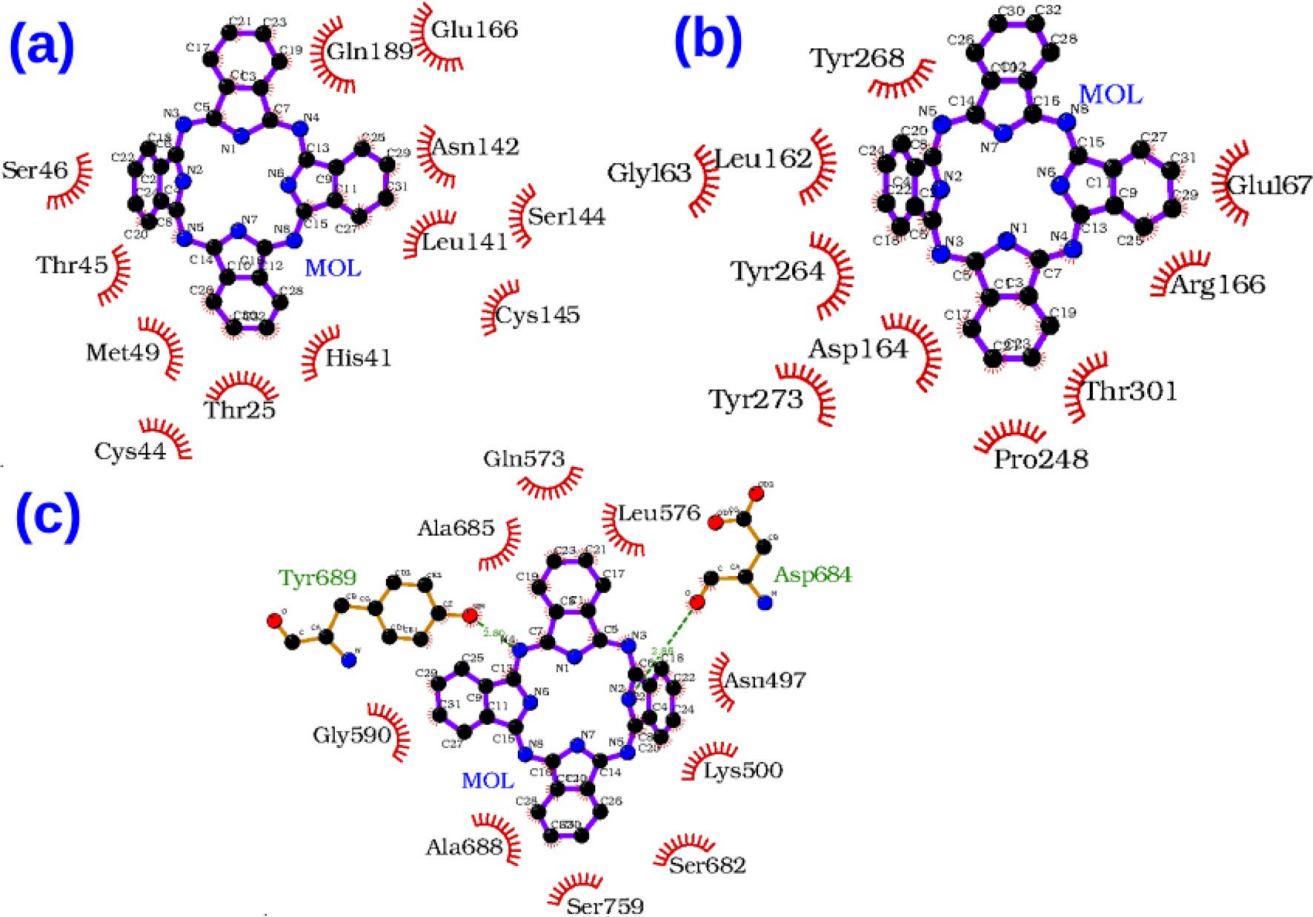
Protein-ligand interaction diagrams for the compound, phthalocyanine with three targets (**a**) 3CLpro, (**b**) PLpro, (**c**) RdRp respectively.

It is desirable to target multiple targets associated with an infection or disease with relatively low affinity compounds than targeting a single specific target with high affinity binding. It it known that certain targets have tendency to mutate much more easily to gain drug resistance^60^. In this occasion it is generally recommended to subscribe a combination of drugs or drug cocktails where each individual compound has specificity and binding affinity towards a target. Another option is to design a molecule which can inhibit multiple targets simultaneously. Such compounds are designed better using a fragment based approach where each fragment can have specificity for a target and by designing a compound with multiple fragments, this can be achieved. Here, we have explored the Drugbank database with the intention whether there are compounds which can bind to multiple targets of Covid-19 associated virus with significant binding affinity. We have identified a few such compounds based on their binding energies computed using AutoDock Vina for the three targets and listed them in Table 4. In this case, we have not included spike protein as a target as the binding affinity to the interfacial site may not be directly correlated to pharmacological activity. The list surprisingly does not include any antiviral compounds but rather many of them are anticancer compounds (Phthalocyanine, Tadalafil, Lonafarnib, Nilotinib and R-428). To understand why these compounds have the ability to bind to multiple targets, we have computed the protein-ligand interaction diagrams using LigPLOT software^61^. This has been computed for the two top compounds namely DB04016 and Phthalocyanine and the results are presented in Figs. 6 and 7 respectively. Further the subplots a, b and c refer to the targets 3CLPro, PLPro and RdRp respectively. The compound, DB04016 has both aromatic groups and polar functional groups so that it can interact with many hydrophobic residues and polar residues in the catalytic site of different targets (refer to Fig. 6). In addition, number of rotational bonds allow the molecule to adopt different conformations in a target-specific manner. The same also is seen in the case of Phthalocynine as the molecule is quite flexible which allows it to adopt a conformation with maximum binding affinity to a specific target (refer to Fig. 7). Based on these results we suggest that the conformational flexibility is an important feature to consider when we aim to design multi-targeting lead compounds.

### Appraisal of the compounds under consideration for clinical trials and repurposed drugs from other chemical spaces

As of today many compounds from the Drugbank database and other chemical databases have been considered as potential against Covid-19. Recently, it was reported that there are more than 300 drug trials in progress^62^. Here, we checked whether the computational screening approach employed in this study could be used to validate the suitability of those compounds considered for clinical trials. Some of the compounds such as Remdesivir, Chloroquine, Lopinavir, Oseltamivir, Ritonavir, Baloxavir marboxil, Favipiravir, Baricitinib, Darunavir Umifenovir, etc., are under clinical trial for Covid-19 (Table S1). The list only includes organics based compounds in clinical trials. Most of these are antiviral compounds and shown to be active against HIV, influenza or Ebola, SARS-CoV-1 and MERS. Being listed in the Drugbank database, these compounds were already part of our screening using AutoDock Vina but did not show up in the list of high affinity compounds (Table 1). One reason is that the Drugbank database has more potent compounds than those considered for drug trials. Another reason for not showing up as top affinity compounds could be due to limited accuracy of the employed scoring method. It is worth mentioning that rescoring the top 10 compounds using MM-GBSA approach, resulted in the change of ranking (Table 2) as compared to their original ranking obtained from the AutoDock Vina (Table 1). This also indicates that accuracy of the scoring function has a clear impact on the outcome.

The binding energy computed using AutoDock Vina for Remdesivir with RdRp is $-$ 7.2 kcal/mol which is higher (by about 3.4 kcal/mol) than the binding affinity of the best compound, Phthalocyanin obtained from the screening. Further it is having higher binding energy (by about 2.7 kcal/mol) than the second best compound RU85053. Probably, AutoDock Vina based scoring has pushed Remdesivir down in the list of high affinity compounds and this can be a case of “false negative” outcome with this approach. In order to check whether another scoring method has any impact on the ranking of the compounds, the binding energies of Remdesivir and other selected set of trial compounds in three selected viral targets^33,62^ were recalculated using a more accurate MM-GBSA approach (Table 5). The results showed that the binding free energy of Remdesivir ($-$ 36.6 kcal/mol) with RdRp target was second least to that of the top compound, RU85053 (Table 2). In addition, Remdesivir also inhibits 3CLpro and appears to be the best compound in the Drugbank database against this specific target which is quite an interesting observation. The compound has the free energy of binding $-$ 44.4 kcal/mol (Table 5) against this target while this value corresponds to $-$ 43.6 kcal/mol for the best compound (Baloxavir marboxil) obtained from combined AutoDock Vina and MM-GBSA based screening (Table 2). The ability of Remdesivir to inhibit two targets namely 3CLpro and RdRp simultaneously may be a reason why this compound is the most potential compound from the Drugbank database against Covid-19^33,62^. This observation gives high-level confidence on MM-GBSA based scoring and also supports the inclusion of this compound in the clinical trial for Covid-19^33,62^. Further analysis of the binding free energy results obtained using the rescoring approach for the other compounds in Table 5 can provide possible explanation for their potency against SARS-CoV-2 targets. The results indicate that the hydroxychloroquine is not the best inhibitor for any of the proteases of the virus but showed moderate binding affinity for RdRp (Table 5). The therapeutic effect of hydroxychloroquine against many RNA viruses including HIV, influenza, MERS, SARS-CoV-1 is due to interference with the pH dependent endosome-mediated viral entry and thus it is reported to be targeting the human cell rather than the viral targets^63,64^. In agreement to this our study reports negligible binding preference to any of the viral targets. Further our study showed that the compound, Oseltamivir and Favipiravir are not potential compounds for Covid-19 as they only exhibited moderate or negligible binding affinities towards the viral targets when compared to the top compounds obtained from combined AutoDock Vina and MM-GBSA based scoring. The compounds such as Baricitinib and Umifenovir showed superior binding affinity towards PLpro and RdRp respectively, among the trial compounds. Darunavir is also found to be a potent compound as it exhibited moderate binding affinities towards all three targets. It was quite striking that the Favipiravir was recently taken to phase III trial by Glenmark Pharmaceuticals for treating Covid-19. But our study showed less significant binding affinity towards RdRP ($-$ 4.1 kcal/mol). We learn from the literature^65,66^ that Favipiravir is not the active form but rather the phosphorylated form, Favipiravir-RTP is known to inhibit RdRp^65,66^. The binding free energy calculations for Favipiravir-RTP with RdRp showed that it is among the top-three inhibitors for this target (with $\Delta \hbox {G}=-32.0\,\hbox {kcal/mol}$).

**Table 5:** The binding free energies computed using MM-GBSA for the trial compounds against three viral targets namely 3CLpro, PLpro and RdRp. The binding energies are in kcal/mol. The standard errors associated with the binding free energies are in the range 0.1–0.3 kcal/mol. The binding modes for all these compounds were obtained from AutoDock Vina.

Drug	3CLpro	PLpro	RdRp
Remdesivir (DB14761)	$-$ 44.4	$-$ 27.3	$-$ 36.5
Baloxavir marboxil (DB13997)	$-$ 43.6	$-$ 16.0	$-$ 22.4
Hydroxy chloroquine (DB01611)	$-$ 12.1	$-$ 14.7	$-$ 25.8
Oseltamivir (DB00198)	$-$ 15.8	0.0	$-$ 19.8
Favipiravir (DB12466)	$-$ 5.4	$-$ 5.4	$-$ 4.1
Favipiravir-RTP	–	–	$-$ 32.0
Baricitinib (DB11817)	$-$ 17.6	$-$ 36.6	$-$ 13.1
Darunavir (DB01264)	$-$ 27.3	$-$ 25.7	$-$ 22.5
Umifenovir (DB13609)	$-$ 26.9	$-$ 16.8	$-$ 35.

The 3CLPro has been considered as the most attractive target for developing therapeutics for Covid-19. As we have mentioned earlier, many research groups have screened and repurposed compounds from different chemical spaces against this target^39–45^. In order to study the potency of these repurposed compounds when compared to compounds that we have identified from Drugbank database, we have also computed the binding free energies of selected compounds. In particular, the compounds, Carfilzomib, Eravacycline, Valrubicin, Lopinavir, Elbasvir and Ritonavir^44,45^ were included in this study and their binding free energies with 3CLPro target were estimated using sequential molecular docking, molecular dynamics and MM-GBSA approach. The binding free energies and different contributions are shown in Table 6. The study shows that the compounds Elbasvir (with $\Delta \hbox {G}=-40.2\,\hbox {kcal/mol}$), Lopinavir (with $\Delta \hbox {G}=-39.3\,\hbox {kcal/mol}$) and Ritonavir (with $\Delta \hbox {G}=-37.3\,\hbox {kcal/mol}$) in this set have better binding affinity towards this target suggesting that chemical spaces other than Drugbank database such as CheMBL, Pubchem also have potential compounds against the viral targets. It is worth noting that these compounds have 3 kcal/mol higher than the two most potent compounds namely Baloxavir marboxil (with $\Delta \hbox {G}=-43.6\,\hbox {kcal/mol}$) and LY-2090314 (with $\Delta \hbox {G}=-43.3\,\hbox {kcal/mol}$) identified from the Drugbank database in this study. We also would like to mention here that depending upon the scoring functions, the relative binding affinities and the ranking of compounds can be different. So, care should be taken when comparing and analysing relative binding affinities and ranking of compounds reported using different computational approaches.

**Table 6:** Total binding free energies and contributions from vander Waals, electrostatic and solvation energies computed for selected repurposed compounds with 3CLPro target. The free energies and different contributions are given in kcal/mol.

Drug	$\Delta E_{vdw}$	$\Delta E_{elec}$	$\Delta G_{GB}$	$\Delta G_{SA}$	$\Delta G_{binding}$
Carfilzomib	$-$ 46.9	$-$ 4.8	33.8	$-$ 6.0	$-$ 23.9
Eravacycline	$-$ 32.7	$-$ 10.7	28.3	$-$ 4.3	$-$ 19.4
Valrubicin	$-$ 50.1	$-$ 16.4	44.3	$-$ 6.5	$-$ 28.6
Lopinavir	$-$ 50.7	$-$ 22.8	40.8	$-$ 6.6	$-$ 39.3
Elbasvir	$-$ 61.4	$-$ 10.1	38.1	$-$ 6.9	$-$ 40.2
Ritonavir	$-$ 54.5	$-$ 24.1	48.3	$-$ 7.1	$-$ 37.3

### Proposing a drug-cocktail for Covid-19

Drug cocktail is a combination of drugs that have best potency against each of the vital drug targets of the virus. Here, we have considered the three vital targets for SARS-CoV-2. The spike protein is not included here as we cannot relate the binding affinity of the drugs to binding site in the interfacial region to the potency. In Fig. 3b, this site is shown to be occupied by the ligands in green color. Further, the spike protein receptor binding domain is shown to have multiple beta-sheet like secondary structures and the ACE-2 receptor is shown with many helix-like secondary structures. If the drugs have stronger binding affinity for both targets then the protein–protein interaction is increased. But if the binding is specific to any one of the targets, then the protein–protein interaction will be reduced. Overall, the binding affinity of drugs in the interfacial region cannot be directly related to their pharmacological activity. We propose that a combination of Baloxavir marboxil, Natamycin and RU85053 can serve as a suitable drug cocktail for Covid-19 as they can respectively inhibit 3CLpro, PLpro and RdRp. It is worth noting that Baloxavir marboxil is already under consideration in combination with Favipiravir for drug trial by a company Shionogi, Toyama Chemical^33^. Even though our study supports the activity of the former compound, the latter one was not identified to be potent against any of the viral targets (Table 5) as it has much higher free energies of binding ($-$ 4.1 to $-$ 5.4 kcal/mol).

## Conclusions

We have searched for potential active compounds in the Drugbank database for targeting different SARS-CoV-2 proteins such as 3CLpro, PLpro, RdRp and spike protein. For each of these targets, we identified compounds with high binding affinity using a double scoring approach. Some of these compounds are already under trial for the treatment of Covid-19 infection. This clearly demonstrates not only the strength of our strategy based on the combined scoring (AutoDock Vina + MM-GBSA based) but also gives confidence for the use of computational approach-based screening as a starting step for drug repurposing/discovery. Moreover, from the list of the identified compounds we also proposed drug(s) which can be either used individually or in combination, against the virus. We also report the multi-targeting capacity of a few drugs like Phthalocyanine, Tadalafil, Lonafarnib, Nilotinib, Dihydroergotamine and R-428 which have the potential to simultaneously inhibit three viral targets such as 3CLpro, PLpro and RdRp. Further, the study also included the binding energy estimation for various compounds which are currently under drug trials. It is shown that Remdesivir binds to RdRp and 3CLpro with high binding affinity indicating that it can be categorised as a multi-targeting drug. Baricitinib and Umifenovir were found to be compounds with superior target-specific binding while Darunavir is found again to be a multi-targeting drug. It is shown that Hydroxy chloroquine and Oseltamivir are not very active against any of the viral targets and in particular, the former compound has only moderate binding affinity towards RdRp.

The study demonstrates how to repurpose drugs from different chemical spaces such as Drugbank database to swiftly develop therapeutics against Covid-19 like viral infection. The compounds identified can be subjected to experimental (through binding assay studies) and clinical (by administering the lead compounds to patients having infection) validations before subscribing to a wider population. Screening the compounds from Drugbank gives as another advantage that these compounds are already validated for favourable ADMET properties and so clinical validation can be quickly performed. The reliability of the lead compounds generated from computational studies is highly dependent on the accuracy of the scoring functions employed. So, we need to employ accurate scoring methods as the one employed in the current study. We are also currently working on developing triple scoring functions by incorporating QM/MM and QM fragmentation based methods to further improving the ranking of the compounds following a force-field based preliminary ranking.

## Computational methods

In this section, firstly we have described methods used for proposing the three-dimensional structure of the viral targets like PLpro and RdRp. This is followed by the description of the computational screening approach to identify potential compounds against the targets for which the structures are obtained both from the homology modeling and experimental structure elucidation approaches. For a selected set of compounds, we also carried molecular dynamics and free energy calculations using molecular mechanics- generalized Born surface area (MM-GBSA) approach.

### Genome to viral targets

The full genome sequence of severe acute respiratory syndrome coronavirus 2 isolate Wuhan-Hu-1 (Covid-19) (NC_045512.2) was retrieved from the National Center for Biotechnology Information (NCBI) database^67^. We identified the following sequences decoding key targets for virus which are responsible for host cell receptor binding, replication and transcription: spike-protein (YP_009724390.1_3), 3L-main protease(QHD43415_5 ), PLpro (QHD43415_3) and RdRp (YP_009725307.1).

### Homology modelling

PDB structures of PLpro and RdRp of Covid-19 were not available when we started the work. Therefore, homology modelling was performed using the SWISS-MODEL server^27^ with default parameters using automated mode for these two targets. For both targets templates were chosen from SARS-CoV-1. The structure for PLpro is based on the PDB id 5Y3E and for RdRp the PDB structure from 6NUR was used as a template. In the former case, the sequence identity was 83% while in the latter case it was 96.4%. Usually, the models based on templates having above 30% sequence identity are considered reliable. So, high sequence identities of the templates used to build models make the obtained structures very reliable.

### Computational screening of compounds from the Drugbank

The compounds from the Drugbank database were screened against the four key viral targets: 3CLpro, PLpro, RdRP and spike protein. For the 3CLpro and spike protein, the three dimensional structures were taken from PDB database. For the remaining two targets, we used the structures from homology models which is described in the previous section. The current version of the Drugbank database (v5.1.5) contains 13,529 drug entries including 2,630 approved small molecule drugs, 1,371 approved biologics, 131 nutraceuticals and over 6,354 experimental drugs. In particular, we only retrieved the subset of the Drugbank database compounds having three dimensional structure and there are about 8773 such compounds. The structure data file (SDF) format of compounds were retrieved from the Drugbank database(v5.1.5)^16^. and with the use of openbabel software, the three dimensional structure for these compounds have been generated. Further, using the autodocktools, the Gasteiger type charges were generated for all these compounds and for the targets. The compounds were screened against the four targets mentioned above using AutoDock Vina software^68^.

Often protein contains multiple binding sites, therefore, recognition of the optimal binding site in appropriate domain is significant to study the interaction between the protein and the inhibitory compound(s). The spike glycoprotein contains receptor binding domain (RBD) responsible for host cell recognition and binding to the Angiotensin Converting Enzyme 2 (ACE2) mammalian receptors. Moreover, this domain undergoes significant conformational change before binding to ACE-2 receptors. The structures for the spike protein in its free state (referred generally as closed state) and in its prefusion state (open state) have been solved using cryo-EM experiments^24,25^. Since the open state of the RBD domain is responsible for binding to ACE-2 receptors, we have used this conformational state for developing therapeutics. Also the binding site has been restricted to be in the interfacial region of the RBD domain and ACE-2 as the drug is eventually supposed to modulate the protein–protein interaction between these two proteins. Similarly in the case of 3CLpro, the structures are reported for both apo state and for the inhibitor bound state. We have considered apo form as it has certain advantages. In particular, in the bound state, the conformational states of the residues around the inhibitor might have reorganised to maximize interaction with the inhibitor. Therefore, this target structure may not be binding effectively to any other ligands with different molecular volumes. The best approach is to adopt a flexible docking but this is computationally very demanding. In this situation, the apo form of the 3CLpro corresponds to its structure in solution and therefore, can serve as a reliable target for the drug design.

During the molecular docking, the grid box size was chosen so that the binding sites in the domain responsible for key processes for viral life are targeted. The grid box dimension was chosen sufficiently enough so that even larger molecules in the chemical space can be identified during the screening. For example in the case of RdRp, we used a grid box dimension of 40X40X40 (with a grid size of 0.375 Å ). The binding site for 3CLpro has been identified based upon its co-crystallized structure with inhibitor, N3^51^. Similar, the binding site for PLpro has been chosen based on the location of GRM inhibitor as in PDB structure 3MJ5. We used Lamarckian algorithm to identify the best binding modes and poses for the ligands within the binding sites. The molecular docking includes the solvation energy which accounts for the change in free energies due to aqueous environment in which protein-ligand complex is embedded. The scoring function also includes the entropic contributions due to conformational degrees of freedom in the ligand. For each rotational bond, 0.2–0.3 kcal/mol entropic contributions are added to binding free energies; the positive value means a more flexible ligand will bind less strongly with the protein (based on the entropic contribution). We have used the routine protocol for molecular docking where the protein framework is fixed while the ligand is flexible. The ligand flexibility is included through the motion along the rotatable bonds. Other intramolecular structural parameters such as bond lengths and bond angles are not altered during the docking. This procedure is efficient but does not account for the effect due to protein flexibility to the binding affinity. So, for the selected ligands, we have carried out all atom molecular dynamics and the free energies of binding were estimated as an average over a few hundreds configurations.

### Molecular dynamics simulations and free energy calculations

Molecular dynamics (MD) simulations require the force-fields (charges and Lennard-Jones parameters and parameters to describe intramolecular structure) to describe the subsystem interactions and initial structures for the protein-ligand complex systems. The most stable protein-ligand complex structure obtained from molecular docking study has been used as the starting configuration for MD simulations. The ligand structure in its bound state to the target protein has been used for computing the electrostatic potential fitted charges by employing Gaussian16 software^69^. For this set of calculations, we have used B3LYP functional and 6-31+G* basis sets. Thus obtained ESP charges and GAFF^70^ force-field, together describe the interaction of ligand with protein and solvents. For the protein targets, we used FF99SB force-field and the water solvent has been described using TIP3P force-field. The protein-ligand complexes were solvated and neutralized by adding sufficient numbers of counter-ions. Among the four different targets studied, the spike protein is the largest one. Setting-up MD for whole spike protein (including both S1 and S2 domains and the three chains) with a solvent box with 8 Å  cut-off yielded, approximately 400000 atoms. Therefore, the calculations were done for the RBD binding domain of spike protein and ACE-2 receptor. In remaining cases, the whole target proteins were included in the MD simulations. Firstly, a minimization run was carried out followed by MD simulations in an isothermal-isobaric ensemble. The temperature and pressure were set to correspond to ambient condition (300 K and 1 atm pressure). The time step for integration of the equation of motion was 2 fs and initially an equilibration was carried out for 5 ps. Followed by this, production runs for a total time scale of 40 ns were carried out. The convergence of energetics and other structural properties (such as densities) during the production runs has been verified (Refer to Figures S4 and S5 of the supporting information. We have also computed the RMSD, Radius of gyration (Rg) and RMSF for the protein alone using the molecular dynamics trajectories of selected protein-ligand complexes and the results are shown in Figures S1, S2 and S3. The results show that all the systems studied have equlibrated well. All the MD simulations were carried out using AMBER16 software^71^. The 500 configurations selected from the last 5ns were used for the subsequent MM-GBSA calculations. The ligand binding induced structural changes need to be accounted for in the proteins for reliably computing the binding free energies and so we have followed this procedure. The MM-GBSA approach employs an implicit solvent model for computing the solvation energies. The polar solvation free energies were obtained using generalized Born approach while the nonpolar solvation free energies are obtained from solvent accessible surface area. The protein-ligand energies were obtained as the sum of van der Waals and electrostatic energies in this scheme. The free energies of binding which are the sum of these four contributions were computed using the MMPBSA.py script^72^ as implemented in the Amber16 software.

## Supplementary information

Supplementary Information.
