# Supplementary material for: Searching for target-specific and multi-targeting organics for Covid-19 in the Drugbank database with a double scoring approach
Source: Sci Rep. 2020 Nov 5;10:19125. doi: 10.1038/s41598-020-75762-7 (PMC7645721; doi:10.1038/s41598-020-75762-7)
Supplement: Supplementary file 1 — Supplementary Information. [file 41598_2020_75762_MOESM1_ESM.pdf]

Supporting information
for
Searching for target-specific and multi-targeting organics for Covid-19 in the Drugbank database with a double scoring approach

Natarajan Arul Murugan[†,*], Sanjiv Kumar[‡], Jeyaraman Jeyakanthan[γ], Vaibhav Srivastava [‡,*]

† Department of Theoretical Chemistry and Biology,
School of Chemistry, Biotechnology and Health,
KTH Royal Institute of Technology, S-106 91,
Stockholm, Sweden

‡ Division of Glycoscience, Department of Chemistry,
School of Chemistry, Biotechnology and Health,
KTH Royal Institute of Technology, Stockholm, Sweden

γ Department of Bioinformatics,
Alagappa University,
Karaikudi, Tamilnadu, India

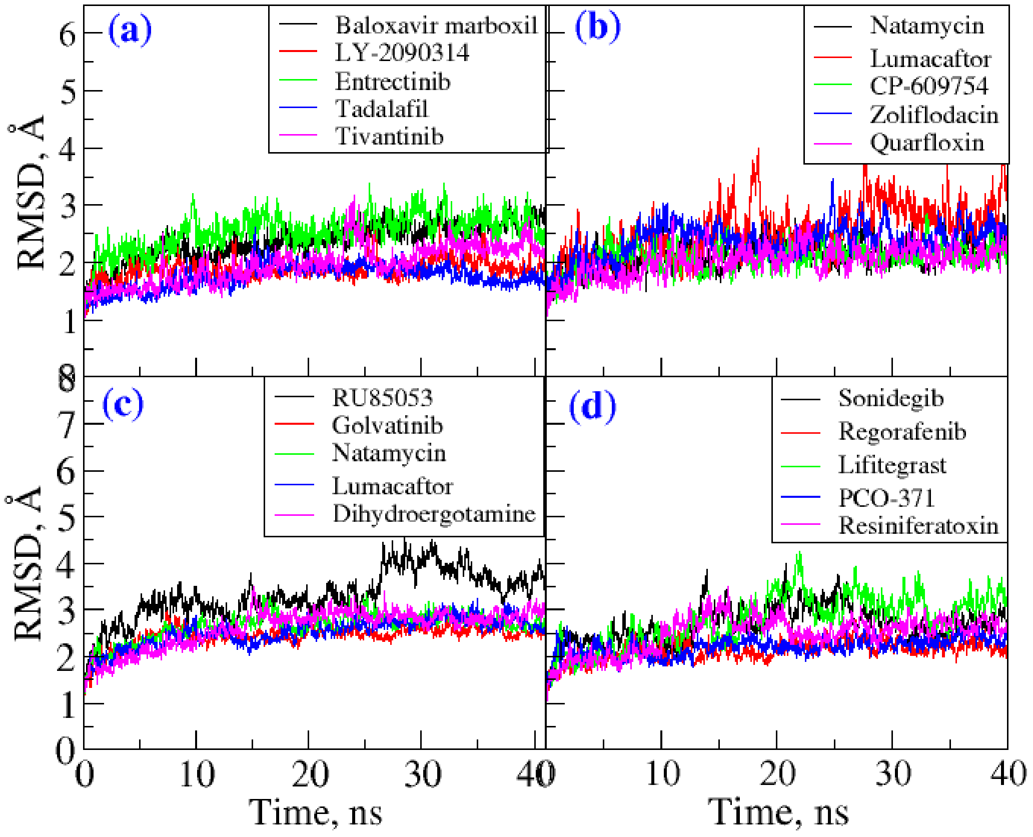

Figure S1 : RMSD computed for proteins in complexes. Only the protein-ligand complexes of top 5 high affinity compounds were considered. Subplots refer to different targets (a) 3CLPro, (b) PLPro, (c) RdRp and (d) Spike protein: ACE-2 complex. The RMSD has been computed in reference to the first configuration in the production run.

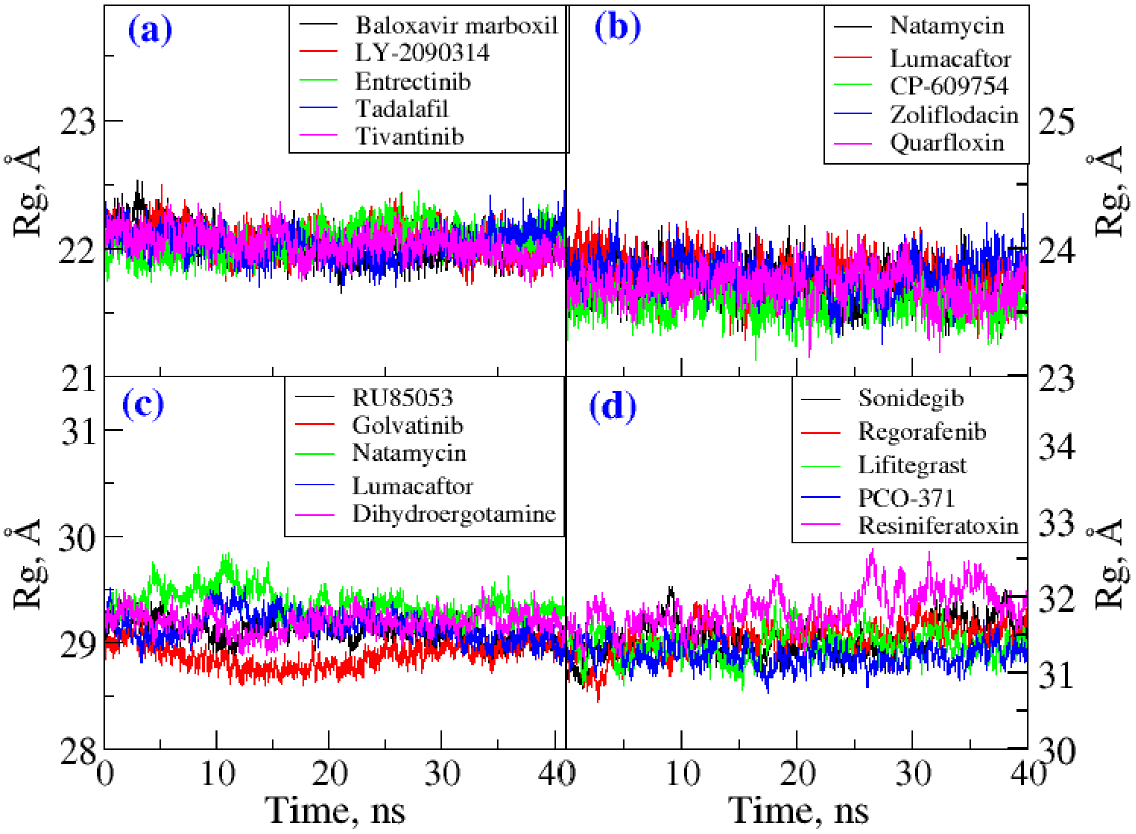

Figure S2: Radius of gyration (Rg) computed for the targets and only the protein-ligand complexes of top 5 high affinity compounds were considered. Subplots refer to different targets: (a) 3CLPro, (b) PLPro, (c) RdRp and (d) Spike protein: ACE-2 complex. The Rg has been computed for the configurations in the production run.

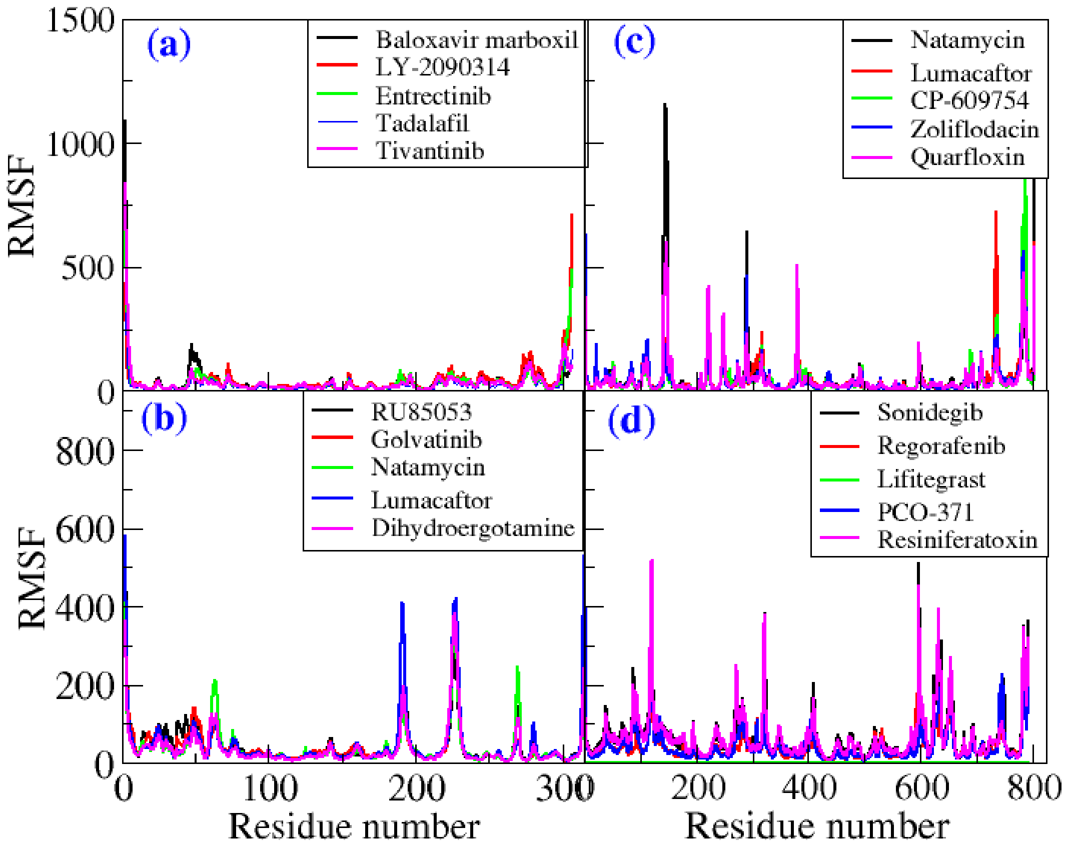

Figure S3: RMSF computed for the targets and only the protein-ligand complexes of top 5 high affinity compounds were considered. Subplots refer to different targets: (a) 3CLPro, (b) PLPro, (c) RdRp and (d) Spike protein: ACE-2 complex. The RMSF has been computed for the configurations in the production run.

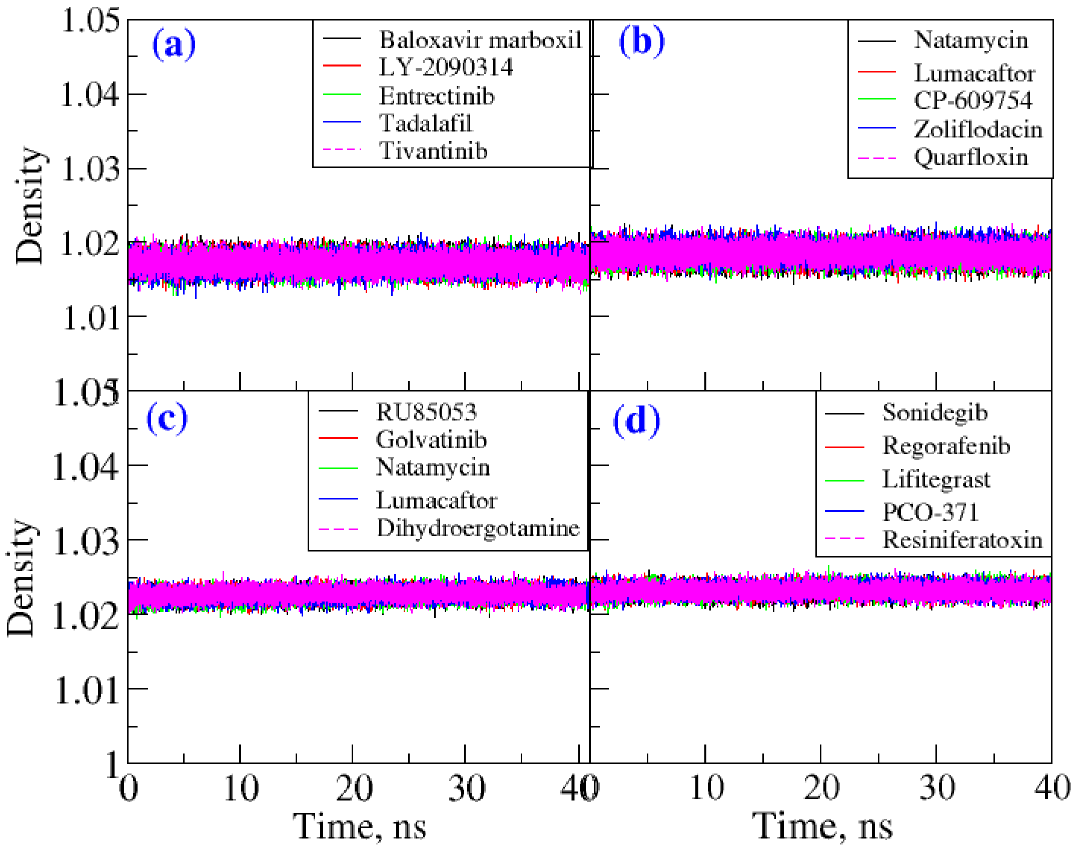

Figure S4: Time evolution of densities of various protein-ligand complexes. Subplots refer to different targets: (a) 3CLPro, (b) PLPro, (c) RdRp and (d) Spike protein: ACE-2 complex. Only complexes of top 5 high affinity compounds were considered for this analysis.

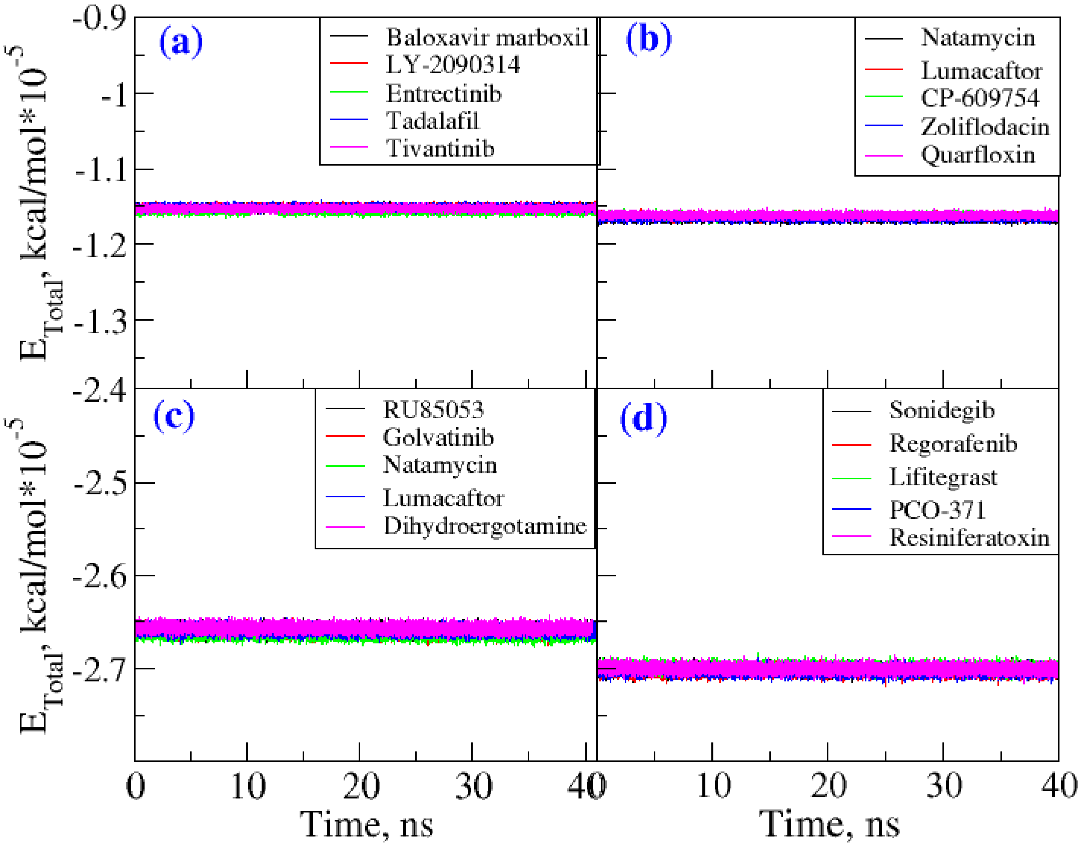

Figure S5: Time evolution of total energies of various protein-ligand complexes. Subplots refer to different targets: (a) 3CLPro, (b) PLPro, (c) RdRp and (d) Spike protein: ACE-2 complex. Only complexes of top 5 high affinity compounds were considered for this analysis.

Table S1: List of compounds in the drug trials for covid-19 therapeutics.

Compound name	3D structure	Company	Category	Mechanism of action
Remdesivir (DB14761)	Yes	Gilead Sciences	antiviral	interferes with the action of viral RdRp
Hydroxychloroquine (DB01611)	Yes	AvKARE, Inc.	antiviral	inhibits stimulation of TLR 9 family receptors
Oseltamivir(DB00198)	Yes	Actavis Group Ptc Ehf.	Anti-viral (influenza viruses A)	neuraminidase inhibitor
favipiravir or Avigan (DB12466)	Yes	Fujifilm Toyama Chemical	Anti-viral (influenza)	inhibition of viral RdRp
Umifenovir(DB13609)	Yes	JSC Pharmstandard	Anti-viral	multiple stages of viral life cycle
Baloxavir marboxil (DB13997)	Yes	Shionogi Co. and Roche AG.	influenza A and influenza B infections	inhibitor of influenza cap-dependent endonuclease
Darunavir(DB01264)	Yes	Teva Pharmaceutical Industries	anti-HIV	nonpeptidic inhibitor of protease (PR)
Baricitinib (DB11817)	Yes	Eli Lilly	Treatment of rheumatoid arthritis	Janus kinase 1 and 2 inhibitor
Lopinavir (DB01601)	No		anti-HIV	antiretroviral protease inhibitor
Ritonavir (DB00503)	No		anti-HIV	protease inhibitor
Azithromycin	No	Pliva	anti-bacterial	Protein synthesis inhibitor
EIDD-2801 (DB15661)	No		Anti-viral	viral error catastrophe
Cobicistat (DB09065)	No	Gilead Sciences And Janssen pharmaceuticals	anti-HIV	inhibitor of human CYP3A proteins
